# Short-term evidence of partner-induced performance biases in simultaneous and alternating dyad practice in golf

**DOI:** 10.1038/s41598-023-48133-1

**Published:** 2023-11-30

**Authors:** Matthew W. Scott, Jonathan Howard, April Karlinsky, Aneesha Mehta, Timothy N. Welsh, Nicola J. Hodges

**Affiliations:** 1 School of Kinesiology, University of British Columbia, War Memorial Gym, 6081 University Boulevard, Vancouver, BC V6T 1Z1 Canada; 2 Department of Kinesiology, California State University, San Bernardino, USA; 3 Faculty of Kinesiology and Physical Education, University of Toronto, Toronto, Canada

**Keywords:** Human behaviour, Social behaviour

## Abstract

Actions in social settings are often adapted based on co-actors. This adaptation can occur because one actor “co-represents” the actions and plans of another. Co-representation can result in motor contagion errors, whereby another’s actions unintentionally interfere with (negatively impact) the actor. In sports, practice often takes place simultaneously or alternating with a partner. Co-representation of another’s task could either harm or benefit skill retention and transfer, with benefits due to variable experiences and effortful processes in practice. Here, dyad groups that either alternated or simultaneously practiced golf putting to different (near vs. far) targets were compared to alone groups (n = 30/group). We focused on errors in distance from the target and expected overshooting for near-target partners paired with far-target partners (and undershooting for far-target partners paired with near-target partners), when compared to alone groups. There was evidence of co-representation for near-target partners paired with far-target partners. We also saw trial-to-trial error-based adjustments based on a partner’s outcome in alternating dyads. Despite differences in practice between dyad and alone groups, these did not lead to costs or benefits at retention or transfer. We conclude that the social-context of motor learning impacts behaviours of co-actors, but not to the detriment of overall learning.

## Introduction

The ability to physically interact with other people in shared environments plays an important role in everyday social interactions. In the domain of sport and exercise, people commonly perform and acquire motor skills in shared environments. These interactions provide unique opportunities for individuals to positively or negatively impact each other’s performance and learning. For example, one may unintentionally move and synchronise with others in an exercise environment, or seamlessly coordinate with teammates in sports.

There is a considerable body of research showing how one’s own actions are influenced by the actions of others, often in undesirable ways (e.g.^1–3^). The observation of another’s action is believed to activate in the observer the same or complementary motor program to the observed action, albeit covertly (e.g.^4–6^). Much of the recent thinking concerning what happens in the brain when one observes others is based on the action observation network (AON;^7^). The AON encompasses what is known as the human “mirror neuron” system^7,8^; brain regions responsible for performing an action, which are similarly activated when observing someone else perform the same action^9^. Accordingly, the AON appears to act as a neurophysiological mechanism allowing the simultaneous (co)representation of another person’s action with the representation of the actor’s own action, enabling the coordination of movements in shared environments^1^.

Behavioural evidence of co-representation has been quantified as imitative tendencies among partners who perform together. This task-based synchronisation has been termed motor contagion^10^, or what we refer to as “task-based” motor contagion. For example, individuals show facilitatory or interfering effects when observed actions are congruent or incongruent with their planned actions, respectively^11^. This imitative tendency is an unintentionally occurring phenomenon, assumed to arise due to the co-representation of an individual’s own motor plan alongside the action (plan) of another^4,12^, even when an observed action is perceived as detrimental or different to the actor’s goal^13^.

One way that behavioural manifestations of co-representation can be investigated is through the measurement of simultaneous actions executed by pairs of individuals (i.e., dyads). When partners sat abreast during a discrete reaching task performed concurrently, partners who could perform an unobstructed straight ahead reach adjusted the height of their reach when their partner reached over an obstacle^14^. Even when a partner’s movements were occluded, height adjustments occurred, indicating that knowledge alone of a partner’s task induces task-based motor contagion. Similar evidence of such contagion has been shown when partners take turns (e.g.^15^). Despite not acting simultaneously, observed actions can still be represented after the observational period, although potential contagion effects may decay^16^. Modulation of these task-based contagions can also occur depending on information available regarding performance outcomes and knowledge of the observed actor’s intentions. For example, after observing an errorful throw that was expected to be accurate, participants adapted for the observed error in their subsequent throwing performance in a compensatory manner^17^. As such, in addition to task-based contagion effects, there are also error-based contagion effects, which manifest as compensatory actions.

Dyad behaviours have also received attention in the context of motor learning, where individuals practice the same or different tasks and are later assessed for retention and/or transfer to new action contexts (e.g.^18–22^). There is some indication that practising in pairs benefits motor learning, as compared to practicing alone^18,19,23^, and various mechanisms for potential benefits have been discussed (see^24^). One explanation for the benefits is that dyad practice provides a combination of physical and observational practice^18^, which can independently and interactively facilitate learning (e.g.^25,26^). Watching a partner perform a similar, yet different task, introduces parameter variability into practice. Although this variability might lead to increased error in practice, it has been shown to aid later retention and transfer^27,28^. Peer observation can additionally promote evaluative processes such as strategy appraisal, social comparisons and error-detection, processes deemed important for motor learning^29,30^. Indeed, Bandura in his social learning theory emphasised how observational learning was impacted by perceptions of ability (i.e., self-efficacy), that mediate later success on the task (e.g.,^31^). Watching others succeed can increase self-efficacy and also what has been termed collective efficacy^32^. However, when individuals have different or competing goals during practice, the impacts on performance and efficacy are less clear. There is a long history showing that the mere presence of another person, be it a partner or bystander, can impact performance in both positive and negative ways (for a recent review see^33^).

Despite explanations concerning how dyad practice might facilitate motor learning relative to practice alone, there have also been studies showing no differences in learning, even when performance effects are evident. For example, partners who simultaneously practised a balance task showed more coordination in their movements during practice and reported more interference from practising together than randomly yoked controls, yet there were no differences in learning outcomes^20^. In a study involving alternating dyad practice of two different types of golf putting actions to the same target, evidence of co-representation was seen, without longer term learning effects^34^. This co-representation manifested as error-based compensatory behaviours in a partner’s subsequent putts; whereby if one partner undershot their target, the other partner would show a tendency to overshoot their target on the next trial. This error-based motor contagion is expected when performers are able to process observed errors in another’s actions, such as in alternating practice contexts^29^.

**Figure 1:**
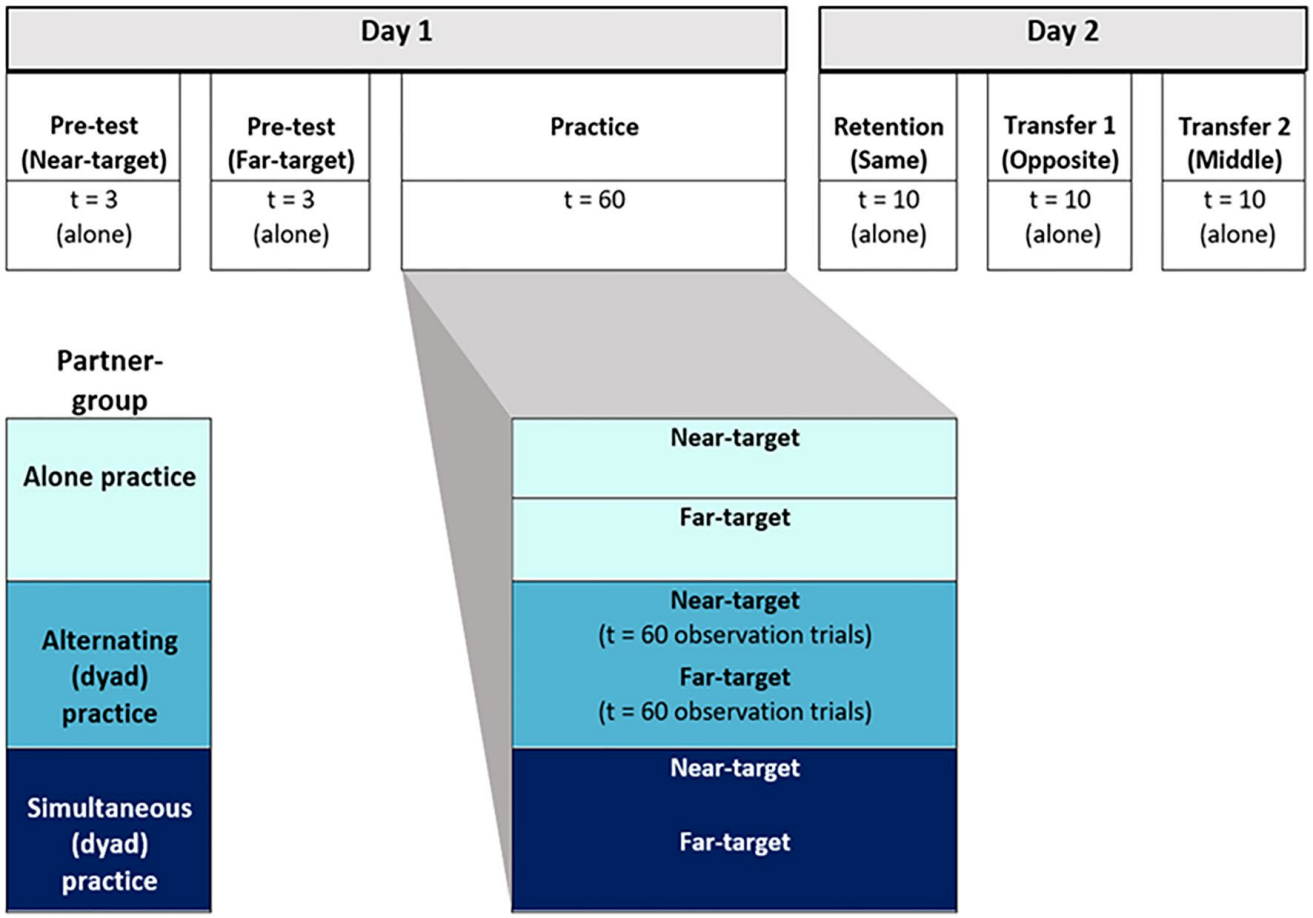
Experimental procedures across all groups. On Day 1, participants first performed a pre-test alone, to near- and far-targets. During practice, participants practiced putting to either a near- or far-target (target-group) and either practised alone or with a partner (partner-group). Dyads always consisted of partners who practiced putting to opposite targets. Partners would either take turns (alternating dyads) or practice concurrently (simultaneous dyads). On Day 2, participants returned alone and first completed a retention test (same target practiced on Day 1) followed by two transfer tests (opposite target and a new middle-distance target).

In the current study, we tested for co-representation and resulting task-based and error-based contagion effects in a dyad practice paradigm. Novice participants practiced golf putting to different distance targets either alone or in pairs. Dyads putted together, either alternating turns or simultaneously putting. Both performance and learning effects were evaluated across practice blocks and next day retention/transfer testing (see Fig. 1). Our primary aim was to test for dissociations between performance and learning as a result of motor contagion in practice that presents as later benefits for learning. A second aim was to test for task-based and error-based contagion effects among partners who putted simultaneously versus alternated turns, where there are differences in the salience of a partner’s actions and errors.

**Figure 2:**
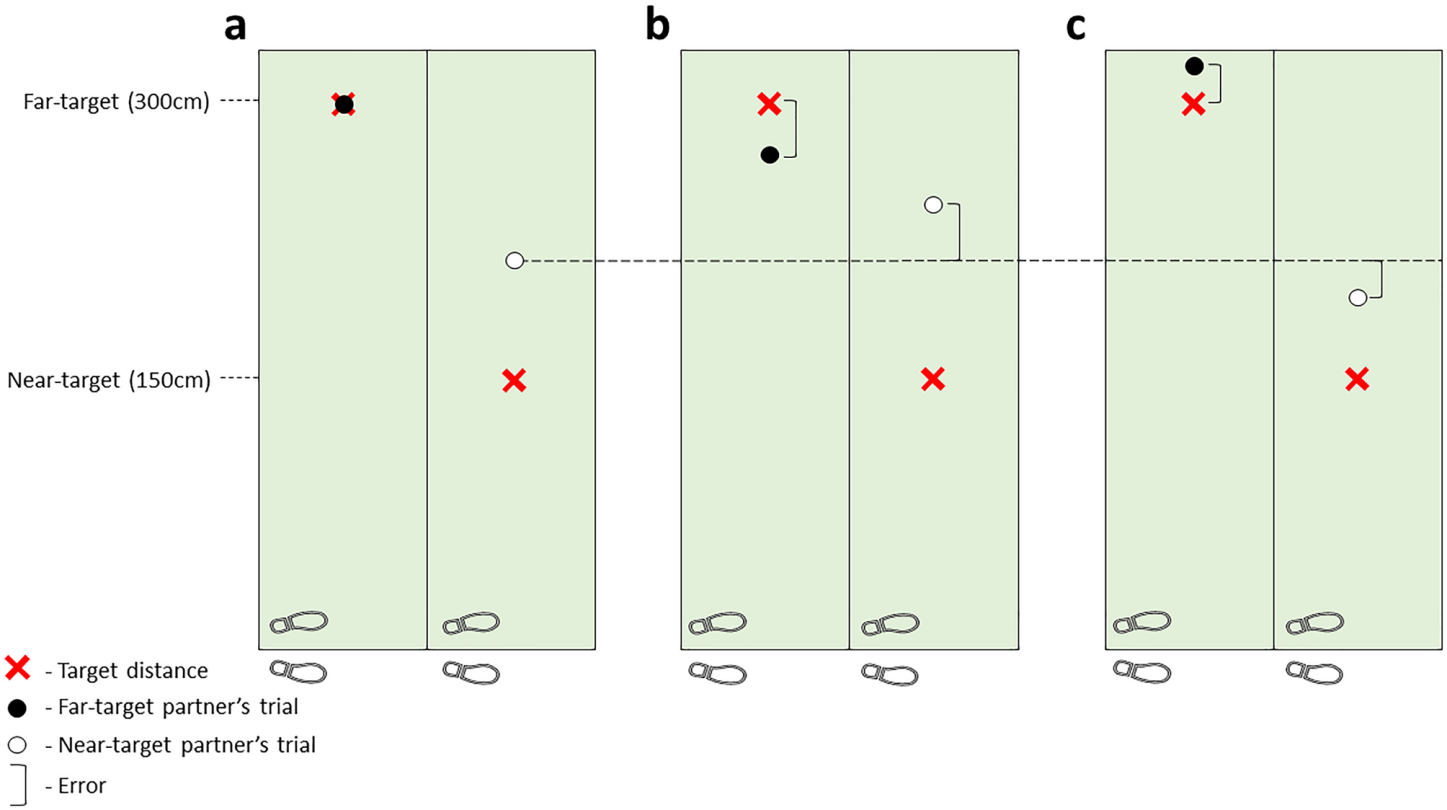
Different types of motor contagion were expected during dyad practice with partners putting to either a near- (150 cm) or far-target (300 cm), shown by the two red crosses. In this example, motor contagion effects are shown for the near-target partner only, in response to performing with a far-target partner. (**a**) Expected task-based motor contagion as shown by the white circle, where near-target partners would show a tendency to overshoot their target in response to co-representation of their far-target partner’s task/performance (black circle). Here the horizontal dashed line originating from (**a**) represents a baseline of task-based motor contagion, to be used as a reference for (**b,c**). (**b**) The situation where we may expect augmented task-based motor contagion due to modulation from error-based processes. In response to seeing their partner undershoot their far-target, the near-target partners would show a greater degree of overshooting to compensate, augmenting the expected task-contagion effect. (**c**) The situation where we may expect attenuated task-based motor contagion due to modulation of error-based processes. In response to seeing their partner overshoot their far-target, the near-target partners would show less overshooting to compensate, attenuating the expected task-contagion effect. These latter modulations shown (**b,c**) would be expected in the alternating dyad groups.

We hypothesized that dyad groups would show evidence of task-based contagion, with errors in the direction of a partner’s target (i.e., constant error, CE) compared to alone control groups (e.g.^14,15,17^). This task-based contagion was expected to be larger in simultaneous groups in comparison to alternating groups as a result of the concurrent representation of both their own and their partner’s task^15,16^. We also expected some trial-to-trial error compensation based on a partner’s previous trial errors (i.e., error-based contagion), which would modulate the size of overall contagion^34^ (see Fig. 2). We expected that the alternating dyads would show evidence of error-based compensation, as between physical trials they are only privy to their partner’s outcome feedback, not their own, so there is more chance that this feedback will influence their next trial^29^. Alternating dyad groups would also be expected to engage in outcome prediction estimation based on their partner’s swing kinematics, which is believed to lead to compensatory-type behaviours in response to errors^17^. This prediction activity would be challenging for simultaneous partners who would not be able to monitor the actions of their co-actor. In addition to modulation of task-based contagion, compensatory behaviours would be evidenced by lag-1 negative correlations within dyads (i.e., between preceding and next trial constant errors).

If the increased variability in experiences and cognitive effort associated with dyad practice facilitates retention processes, then we would expect benefits for dyad groups in comparison to alone groups in retention and transfer testing (evidenced by lower absolute and variable error). However, if the potential contagion effects from practising with a partner remain in retention, then instead there will be costs associated with dyad practice evidenced by larger errors. In addition to physical performance measures, we assessed psychosocial indices related to motivation and competence/self-efficacy, as well as task engagement. We predicted that dyad groups would report greater interest/enjoyment^24^, devoting more effort to the task than alone groups and that dyads would show higher competence than alone participants associated with observational practice of their partner. However, we did expect competency judgements to be moderated by the task goal. Far-target partners were expected to perceive their partner as more competent than themselves as a result of their partner being more accurate (because of the easier near-target). Due to the social nature of the situation, we expected dyads to be more engaged in the task than the alone participants, potentially enhanced for alternating dyads who could better attend to their partner than simultaneous dyads.

## Results

We tested for partner-group differences across all phases of the study using preplanned orthogonal contrasts (alone groups vs. the dyad groups and alternating vs. simultaneous dyad groups; see Methods for full details). Descriptive statistics and results for pre-test data and full outputs of linear mixed effects (LME) models are given in Supplementary Materials.

### Outcome analyses

#### Practice

##### Constant error (CE)

**Figure 3:**
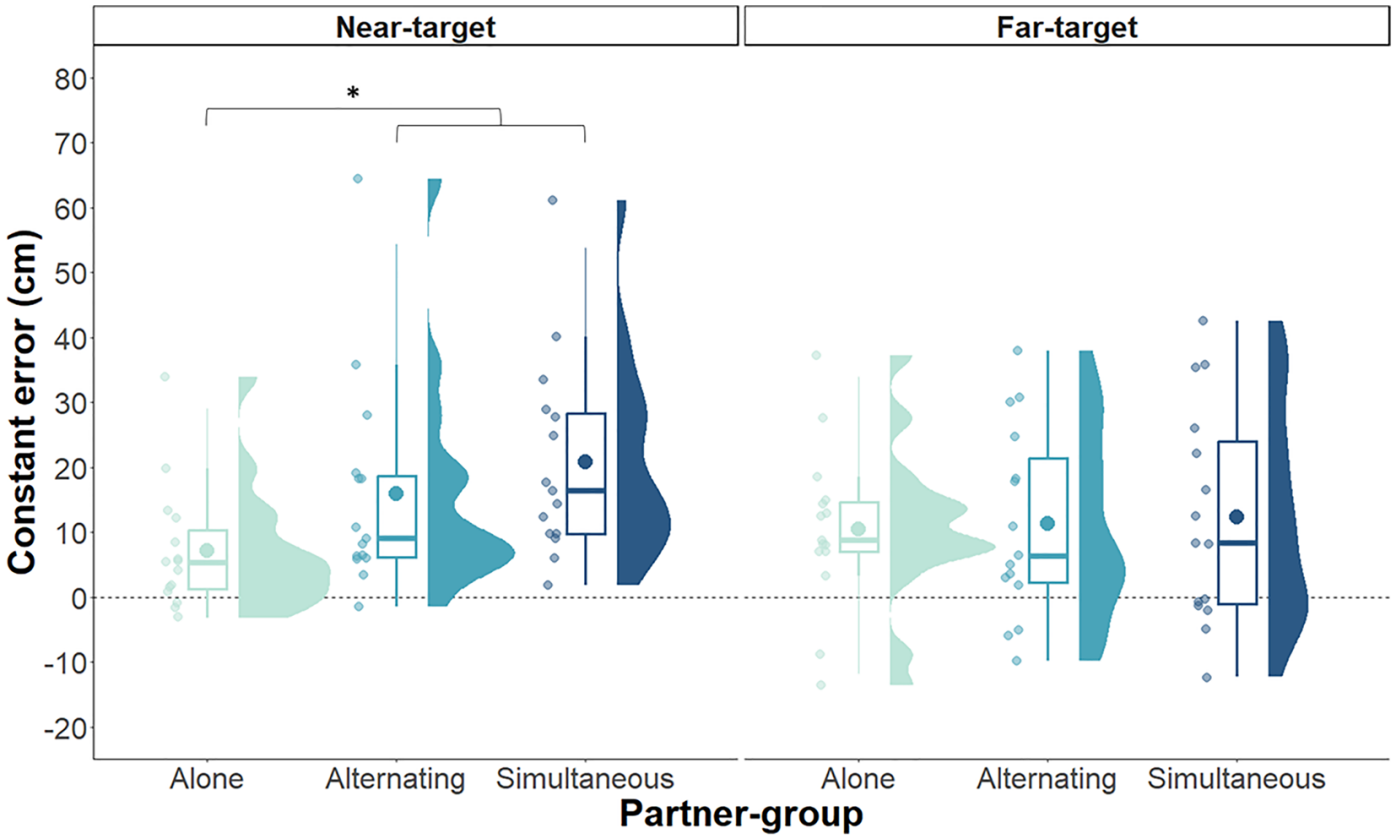
Constant error (CE) in the Y direction collapsed across practice blocks for near-target (left panel) and far-target (right panel) subgroups. Individual data points of jitter plots depict participant means. Boxplots represent median and quantiles, with points within boxplots representing group means. Density profiles are also shown representing the distribution of scores for participant means. Values above the dashed line (0 intercept on the y-axis) show overshooting on average, whereas values below the dashed line represent undershooting on average. *p < 0.05.

We were most interested in the CE data to test for contagion effects in the partner-groups. Data were analyzed separately for the near- and far-target subgroups due to different predictions regarding directional bias. LME models without the interaction term between partner-group and practice block had the best model estimates. For the near-target subgroups, there was a significant effect of block, *β* = −4.05 cm, *p* < 0.001, reflecting a decrease in error across time. As illustrated in Fig. 3, alone and dyad subgroups differed significantly throughout practice (*β* = −11.23 cm, *p* = 0.01). As predicted, overshoot errors were higher in the dyad subgroups (*M* = 18.36 cm, *SD* = 53.22) than alone group (*M* = 7.13 cm, *SD* = 43.27). There was no difference between the alternating and simultaneous dyad subgroups (*β* = −5.00 cm, *p* = 0.32). For the far-target subgroups, directional errors did not significantly change across blocks (*β* = −1.80 cm, *p* = 0.25) and there were no differences between the alone and dyad subgroups (*β* = −1.31 cm, *p* = 0.77), nor the alternating and simultaneous subgroups (*β* = −1.09 cm, *p* = 0.83).

**Figure 4:**
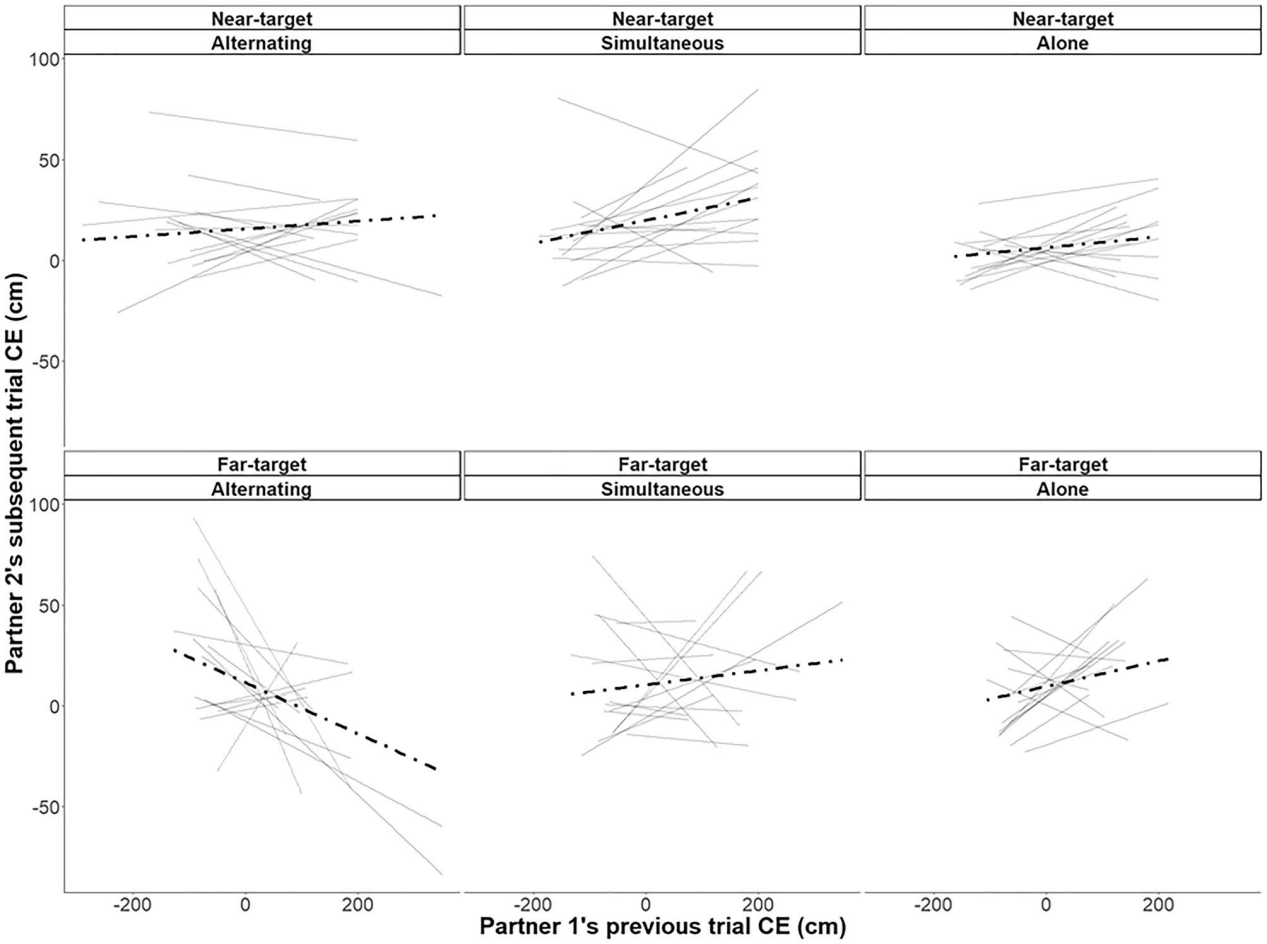
Partner effects for near-target (top row) and far-target (bottom row) practice subgroups, for alternating dyads (left panels), simultaneous dyads (middle panels), and for randomly yoked alone “dyads” (right panels). The y-axis represents a dyad member’s constant error (CE) on a given trial and the x-axis represents their partner’s CE on the previous trial. Regression lines are plotted to show the relation (i.e., partner effect) between these variables for each participant of the subgroups (solid lines), with dashed regression lines representing the average partner effect for the entire subgroup.

A trial-to-trial analysis was conducted on the CE practice data, looking at within-dyad partner effects as a function of group. We expected negative relationships between partners in the alternating dyad group, reflective of error-based compensations. As illustrated in Fig. 4, there was a negative correlation for alternating dyads, but only for partners in the far-target subgroup. When we ran statistical analyses on these data, there was a significant interaction between these trial-to-trial partner effects and partner-group. A partner’s previous trial was negatively associated with their partner’s next trial in the alternating group but not in the simultaneous group (*β* = −0.12 cm,* p* = 0.002). Although this negative relationship appeared to be driven by the alternating far-target group, the three-way interaction between partner group, partner effect and target group was not significant (*β* = 0.08 cm,* p* = 0.08). When we compared randomly yoked-alone group partners to the dyad groups, there was not the expected partner effect X partner-group interaction (*β* = 0.05 cm,* p* = 0.17), but there was a three-way interaction between partner-group, partner effect and target-group (*β* = −0.09 cm,* p* = 0.02). On average, partner effects were more negatively associated with subsequent error for the far-target partner of dyads than in the randomly-yoked alone group. In the dyad groups, the far-target partners showed compensatory behaviours in response to their near-target partner’s previous trial error, which was primarily driven by the alternating group partners.

##### Absolute (AE) and variable error (VE)

**Figure 5:**
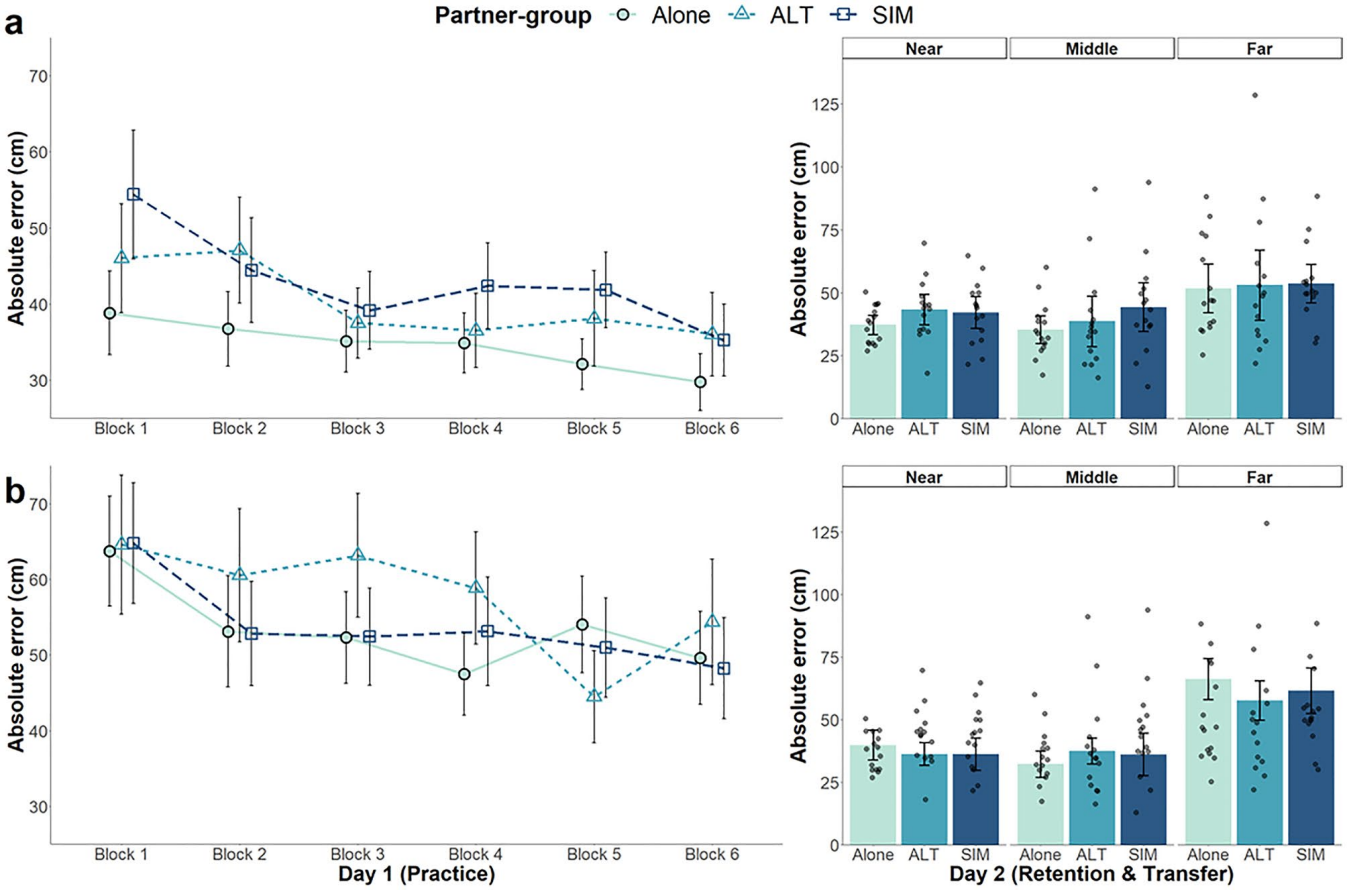
Mean absolute error (AE) across practice and Day 2 retention/transfer tests for partner-groups (alone, *ALT* alternating, *SIM* simultaneous). Near-target subgroups are shown in (**a**) and far-target subgroups are shown in (**b**). Each block during practice represents 10 trials. On Day 2, error for each partner-group is presented for each target (near, middle, far). Individual data points represent participant means. Error bars represent 95% confidence intervals.

**Figure 6:**
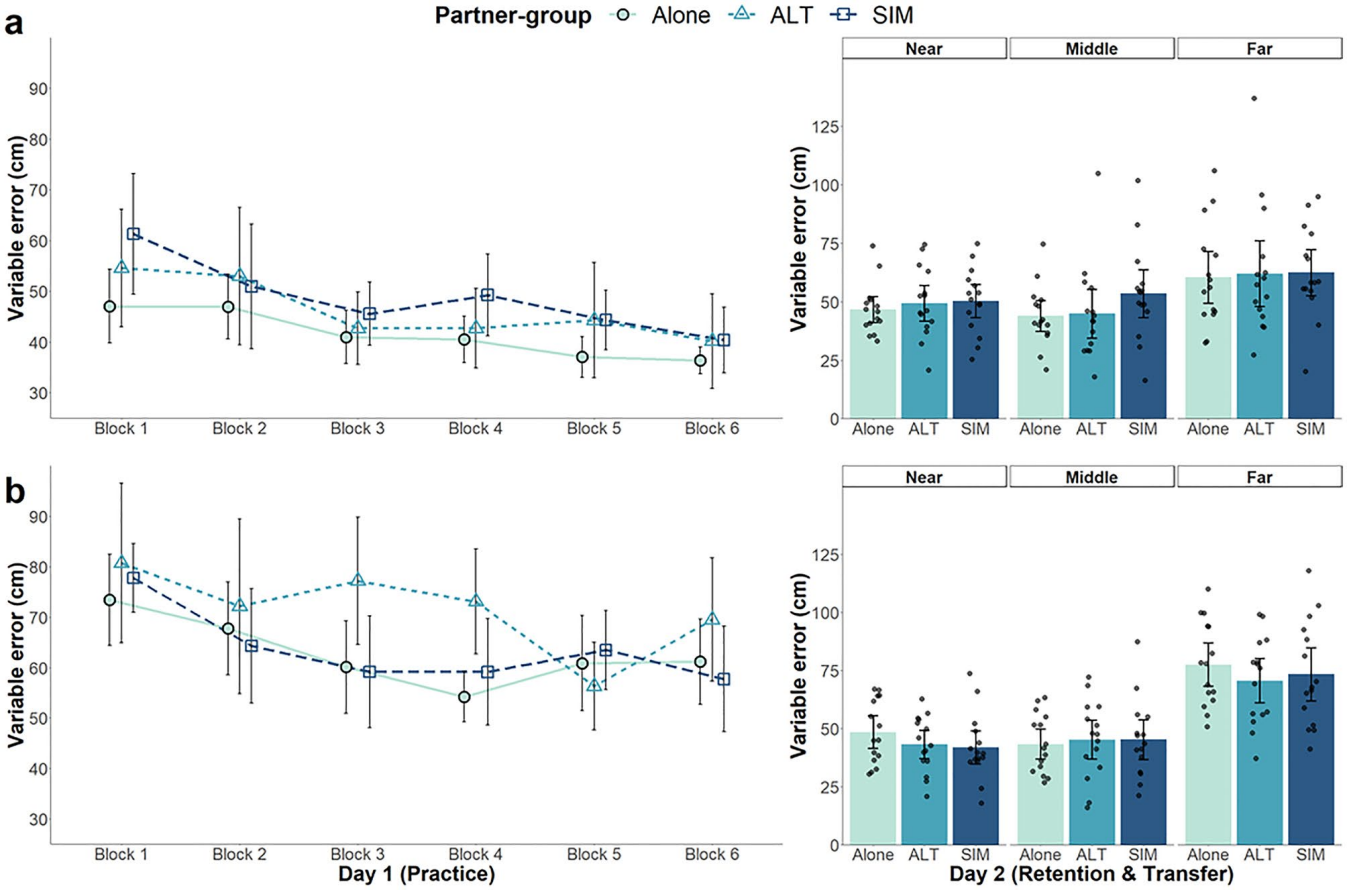
Mean variable error (VE) across practice and Day 2 retention/transfer tests for partner-groups (alone, *ALT* alternating, *SIM* simultaneous). Near-target subgroups are shown in (**a**) and far-target subgroups are shown in (**b**). Each block during practice represents 10 trials. On Day 2, error for each partner-group is presented for each target (near, middle, far). Individual data points represent participant means. Error bars represent 95% confidence intervals.

To provide an overall indication of performance in practice, we also analyzed AE and VE (Figs. 5 and 6, respectively). For AE, a model without interactions for fixed-effects provided the best model fit. Far-target subgroups had more error than near-target subgroups (*β* = 15.49 cm,* p* < 0.001). With respect to our main partner-group manipulation, the alone group did not differ from the dyad groups (*β* = −3.99 cm, *p* = 0.13) and there were no differences between the dyad groups (*β* = 0.69 cm,* p* = 0.82). There was a general decrease in error across practice blocks (*β* = −4.07 cm,* p* < 0.001), with participants becoming more accurate with practice. VE showed a similar pattern as AE data. Within-block variability was greater for the far- versus near-target subgroups (*β* = 20.59 cm,* p* < 0.001). There were no differences between the alone and dyad groups (*β* = −5.12 cm,* p* = 0.09), nor between the dyad groups (*β* = 3.03 cm, *p* = 0.31). Participants decreased VE over time as a function of practice block (*β* = −4.89 cm,* p* < 0.001; Fig. 6).

#### Retention and transfer

##### Absolute error (AE)

AE retention data are illustrated on the right of Fig. 5. LME comparisons indicated a model consisting of interactions between partner-group and target (near, middle, far) and between target-group and target was the best fit. There were no differences in error between the near- and middle-targets (*β* = 1.80 cm,* p* = 0.29), but there was more error for the far- versus near-target (*β* = 19.96 cm,* p* < 0.001). Although there were no overall group differences involving partner-group (alone vs. dyads, *β* = 0.73 cm,* p* = 0.75; alternating vs. simultaneous, *β* = −1.76 cm, *p* = 0.53), there was a significant interaction between partner-groups (alone vs. dyads) when comparing the near- to the far-target (*β* = 7.82 cm, *p* = 0.03). Only the alone group showed significant differences in error between the near- and far-targets. There were no target related differences between the dyad groups (*β* = −0.18 cm,* p* = 0.97). There were also no partner-group related differences when comparing the near- to the middle-target (alone vs. dyads, *β* = 4.39 cm, *p* = 0.22; alternating vs. simultaneous, *β* = 2.71 cm,* p* = 0.51). There was no target-group effect (*β* = 0.07 cm, *p* = 0.75), but there was an interaction between target-group and the near- versus far-target (*β* = 13.13 cm,* p* < 0.001). Surprisingly, for the far target only, it was the far-target subgroups (Fig. 5, panel B) that had more error than the near-target subgroups. There was no target-group interaction when comparing the near- to the middle-target (*β* = −0.06 cm, *p* = 0.85).

##### Variable error (VE)

A similar pattern of results was seen for VE as for AE, except there were no partner-group interactions (right side of Fig. 6). There were no differences in between-trial variability for the alone vs. dyad groups (*β* = −0.09 cm,* p* = 0.97), nor alternating vs. simultaneous dyads (*β* = −1.91 cm,* p* = 0.56). The target-groups also did not differ on Day 2 tests (*β* = 1.66 cm,* p* = 0.53). Comparison of the targets revealed no significant difference between the near- and middle-target (*β* = 0.62 cm,* p* = 0.77), only between the near- and far-target (*β* = 21.73 cm,* p* < 0.001). There was also a significant interaction between target-groups when comparing the near- and far-target (*β* = 14.98 cm,* p* < 0.001), but not between the near- and middle-target (*β* = −1.41 cm,* p* = 0.74). The interaction was due to the near-target subgroups (Fig. 6a), having less error than the far-target subgroups (Fig. 6b), for the far-target in retention.

### Psychosocial measures

#### Intrinsic Motivation Inventory (IMI)

Descriptive statistics for the IMI subscales are shown in Table 1. For the interest/enjoyment subscale, the simultaneous group reported higher levels of interest than the alternating group (*β* = −1.05, *p* = 0.028), but there was no difference between dyads and the alone group or a target-group main effect. The alone versus dyad interaction with target-group was significant (*β* = 1.41, *p* = 0.02). Near-target dyad partners reported greater enjoyment/interest than the alone near-target subgroup; this relationship was reversed for the far-target subgroups. There was no interaction between the two dyad groups and target-group.

**Table 1:** Group mean ratings (and between-participant SDs) for each subscale of the Intrinsic Motivation Inventory.

Target-group	Partner-group	Intrinsic Motivation Inventory subscale				
		Interest/enjoyment	Perceived competence	Partner’s competence	Pressure/tension	Effort
Near-target	Alone	3.83 (1.59)	3.41 (1.38)	−	2.61 (1.28)	5.25 (1.15)
	Alternating	4.02 (1.05)	3.65 (1.00)	4.23 (1.21)	2.75 (1.32)	4.83 (1.48)
	Simultaneous	5.07 (1.43)	4.60 (1.24)	4.17 (.98)	2.85 (1.37)	5.37 (1.3)
Far-target	Alone	4.98 (1.25)	3.89 (.67)	−	3.53 (1.58)	5.11 (1.33)
	Alternating	4.18 (1.23)	3.24 (1.08)	4.76 (1.2)	2.92 (1.18)	4.84 (.84)
	Simultaneous	4.38 (1.07)	3.37 (.78)	4.93 (.96)	2.71 (1.05)	4.73 (1.28)

Dyads reported greater levels of perceived competence than alone participants (*β* = −0.71, *p* = 0.04). Comparison between the dyads revealed greater perceived competence for the simultaneous than the alternating group (*β* = −0.95, *p* = 0.02). There were no differences between target-groups, but there was an interaction for the comparison between the alone and dyad groups and target-group (*β* = 1.30, *p* = 0.007). The near-target dyad subgroups reported greater perceived competence (*M* = 4.12, *SD* = 1.12) than the alone near-target subgroup (*M* = 3.41, *SD* = 1.38), but this relationship was reversed in the far-target subgroups (Dyads: *M* = 3.31, SD = 0.93; alone: *M* = 3.89, SD = 0.67). There were no partner-group or target-group effects for pressure/tension, partner competence, or effort (see Supplementary Table S9).

#### User Engagement Scale (UES)

Group means and standard deviations for UES subscales are presented in Table 2. The only group difference was for endurability. There was a significant interaction when comparing the alone and dyad groups with target-groups (*β* = 1.09, *p* = 0.049). Dyads in the near-target subgroups (*M* = 4.60, *SD* = 1.24) had higher ratings than the alone near-target subgroup (*M* = 3.91, *SD* = 1.41) but the reverse was true in the far-target subgroups (Dyads, *M* = 4.18, *SD* = 1.01; Alone, *M* = 4.58, *SD* = 1.33). See Supplementary Table S10 for statistical output for all subscales.

**Table 2:** Group mean ratings (and between-participant SDs) for each subscale of the User Engagement Scale.

Target-group	Partner-group	User Engagement Scale subscale				
		Perceived usability	Novelty	Felt involvement	Focused attention	Endurability
Near-target	Alone	5.22 (1.41)	3.93 (1.61)	4.38 (1.28)	3.13 (1.21)	3.91 (1.41)
	Alternating	5.32 (1.11)	3.98 (1.16)	4.35 (1.18)	3.73 (1.24)	4.18 (1.12)
	Simultaneous	5.61 (1.36)	5.11 (1.04)	5.47 (1.31)	4.03 (1.49)	5.02 (1.36)
Far-target	Alone	5.11 (1.33)	4.93 (1.5)	4.78 (1.27)	3.83 (1.19)	4.58 (1.33)
	Alternating	5.25 (1.07)	4.47 (1.16)	4.82 (1.23)	3.69 (1.28)	4.18 (1.06)
	Simultaneous	5.54 (.97)	4.56 (1.04)	4.92 (1.05)	4.03 (1.01)	4.18 (.97)

## Discussion

Our primary aim was to determine whether partners would impact the golf putting performance of each other during practice putting to different targets and if so, whether this would impact later measures of learning compared to alone groups. We compared dyad groups that putted simultaneously or alternated turns to assess impacts of viewing a partner’s action kinematics and evaluation of outcome errors on any potential contagion-type effects. For the dyads, there was evidence of partner influences in practice, which manifested as task-based motor contagion—significantly greater overshooting for near-target dyad subgroups compared to the alone subgroup. This tendency to overshoot did not differ significantly between the two dyad subgroups and did not manifest as greater absolute error in practice nor in measures of learning. This lack of difference between the dyad subgroups suggests that the overshooting was driven by co-representation of the partner’s task^14^ and not the observation of swing kinematics *cf*.^17^. However, there was also evidence for error-based contagion behaviours in the alternating dyad group for far-target partners, which may have modulated any task-based contagion effects. Far-target partners adjusted their performance in a compensatory fashion, showing negative relationships between their own and their partner’s error on the previous trial. In the following paragraphs we will unpack these data and provide potential explanations.

As expected, analysis of the CE data during practice revealed significantly greater overshooting for near-target partners of dyads compared to the alone near-target subgroup. This finding aligns with previous dyad research showing task-based motor contagion effects^14,15,17^. The target goals and potential outcomes of a partner appear to unintentionally impact the behaviours of a partner even though the goals of the task are distinct. During practice, despite a trend for greater overshooting for the simultaneous near-target subgroup, there was no statistical difference between the dyad groups. As such, these directional biases appear to be driven by co-representation of the partner’s goal, not vision of action kinematics, as only the alternating group had opportunities to observe each other’s kinematics. van der Wel and Fu^14^ not only found a task-based motor contagion effect when participants could see one another, but also when their partner was occluded. In the context of our findings, the knowledge of a partner’s goal and outcome was enough to induce co-representation of their partner’s performance. This co-representation manifested as a directional bias, presumably due to interference at the action planning phase.

There was also some evidence of error-based motor contagion effects in the alternating group, which were compensatory. These compensations based on target error might explain some of the reduced task-based contagion effects in the alternating versus simultaneous groups. Although limited to far-target partners, there was reduced error in response to seeing their near-target partner overshoot their target and increased error in response to seeing their partner undershoot. This compensation is congruent with previous literature where partners adjusted for each other’s error (e.g.,^34–36^). The content and saliency of outcome feedback about one's own performance and that of a partner differed across the two partner-groups, potentially explaining these error-based contagion differences. There is considerable evidence showing the importance of augmented feedback for motor learning (e.g.,^29,37^, for a recent review see^38^), as well as for enhancing observational learning (e.g.,^39^). The alternating group saw partner kinematics and performance outcomes in between their physical trials. Thus, not only would the partner’s performance be more likely to influence the observer’s next trial^29^, being the most recent source of information preceding their physical trial, but the observer would also be more likely to engage in sensory predictions about their partner’s actions, with errorful trials potentially leading to compensation-type contagions^12,17^. In contrast, simultaneous dyads performed concurrently and received self-feedback promptly before their next trial. They also had limited-to-no vision of their partner’s kinematics, given that near-target partners had their back to their partner and far-target partners had only limited peripheral vision. As such, the absence of any error-based compensation in the simultaneous dyads was expected.

Corrective behaviours in response to errors in a partner’s trial may be more common in response to partners who show relatively stable and lower error performance (i.e., the near- vs. far-target partners). In these cases, target misses are more salient and hence produce “prediction-error” based implicit motor corrections^17^. Ikegami et al.^17^ showed that experts were susceptible to task-based contagion after observing variations in action outcomes that were presumed to be intended. Conversely, when participants observed actions where the outcome deviated from the known/intended target, compensatory behaviours were instead exhibited (error-based contagion). Two key differences between the current study and that of Ikegami et al.^17^ were that here we tested novices and also provided outcome feedback on execution trials. Experts have established motor repertoires and presumably well-developed sensory predictions. Our data give some tertiary evidence that even in the absence of established sensory-motor models, prediction errors are possible and lead to compensatory adaptations in novice performers. Moreover, even though our participants were able to correct for any partner contagion errors in their own performance as they received outcome feedback, there was still evidence for task-based contagion. It is likely that if we had prevented feedback, these error-based contagion effects would be exacerbated.

We conducted this study to test for potential dyad effects in retention, beyond the initial exposure when practising together. We predicted that practice with a partner with different target-distance parameters would facilitate retention and transfer due to increased visual experience of different parameters during practice (e.g.,^28,40^). Moreover, we expected that the additional effort required to stave off interference effects from a partner would positively impact processes beneficial for learning, such as greater elaboration and contrasting^41,42^. Despite our predictions, there were few differences between the groups on measures of retention and transfer. Although the dyad groups had lower error on the far-target compared to the alone group in retention/transfer, this was not the case for the near-target, with dyad groups showing more error (they did not differ for the non-practised middle-distance transfer target). The additional experience from practise with a partner was not enough to translate to beneficial transfer effects, even though such effects have been seen in observational learning studies^27,43^. This lack of transfer may be because there was only exposure to one additional parameter variation during practice, rather than several as is typically the case when variable versus constant practice effects for motor learning emerge^44^. Moreover, the lack of any group differences suggests that any observed “interference” effects in practice were not at a level sufficient to cause lasting consequences.

The lack of difference between alone and dyad groups in measures of retention could also be a result of multiple mechanisms working simultaneously to impact performance and learning^24^. The mere presence of another individual can bring about social facilitation or inhibition effects, depending on the learner’s skill as well as the precision requirements of the task^33^. Even though partners had different goals, just being in a dyad could promote competition, which may undermine future learning^22^. Although we took subjective measures of motivation, competence and engagement, as discussed below, we did not include measures of competitiveness. Control of such factors is needed in future research to better understand how and when dyad practice may benefit motor learning.

One unexpected result regarding retention and transfer were target-group specific effects. The near-target subgroups showed better transfer than far-target counterparts (for AE and VE), most notably for the far-target. We would have assumed that far-target partners would be more accurate on the far-target than near-target partners due to constant, task-specific practice^45^. While not to speculate too much about non-hypothesized target-related effects, it is possible that the benefits seen for the near-target subgroups were due to increased stabilization associated with progressing from easier to more difficult conditions (rather than the reverse). The errorless learning framework might lend itself as a potential explanation for this benefit. Accordingly, easier practice conditions promote a more implicit type of learning, which leverage automatic rather than conscious processing, making learning more robust over time^46^. It cannot be confirmed, however, whether the reduced errors alone contributed to these improvements to a more difficult target (and there were no differences for the medium distance target).

Improved transfer for the near-target subgroups may also be a result of enhanced psychosocial processes associated with practice to an easier target, such as greater perceptions of competence especially in the presence of a more errorful (far-target) partner. Near-target dyad partners rated themselves higher for perceived competence compared to their alone counterparts. In line with social learning theory^31^, seeing their partner being more errorful and receiving knowledge of their own and their partner’s error, appears to have promoted evaluative comparisons between partners, making near-target partners perceive themselves as more competent (i.e., increased self-efficacy)^47^. These comparisons and related perceptions may have also led near-target partners of dyads to rate the task as more interesting/enjoyable (reflecting intrinsic motivation^48^) than their alone group counterparts (and the opposite for far-target subgroups). This trend further extended to the endurability subscale of the UES. Endurability provides an indirect measure of perception of success given it is the likelihood of doing the task again or recommending it^49^. Near-target partners of dyads reported higher competence in the task and were more willing to do the task again. Together, these data highlight the contribution of self-other comparisons on psychosocial aspects of dyad learning, which in this study were dependent on target-group.

Although there has been research showing beneficial learning effects associated with practising in pairs^18,19,23^, there have also been a number of studies failing to show benefits^20,34^. Here, we did not show any learning benefits compared to alone practice, but practising with a partner did impact on the partner’s short-term performance and dyad practice was generally perceived as more enjoyable and promoted increased task competence. When learning new skills, particularly individually performed skills, coaches may create a more engaging practice environment by instructing individuals to practice with others^24^. Congruent with other dyadic learning literature, our data suggest that even though partners are a potential source of interference, individuals will learn as efficiently as when learning alone and there is the potential for enhanced learning. Note, however, that our target distance manipulation within dyads was designed to induce motor contagion-like behaviours. Allowing partners to practice tasks with the same target parameters may influence the competitive or cooperative nature of the environment, potentially providing greater opportunities for learning^22^. However, to further understand the dynamics involved in dyadic learning and improve the application of this research, future investigations should involve more ecologically valid scenarios. This might involve allowing partners to make practice decisions and self-determine how to organize shared practice time in order to provide insight to preferred practice and information sharing behaviours.

In our work, we have only studied unintended consequences of performing with a partner in a sport-type context, where one partner’s performance outcome has no direct consequences for the other partner. Golf, in this aspect, is unique, because in an actual round of golf, a performer’s target distance changes depending on where the ball lands and these locations and required shots are likely to vary between competitors (dyads or groups). Collectively, our findings and those of Ikegami et al.^17^ suggest that experienced golfers would show performance variability due to observing their competitor perform, or just simply knowing the distance they are putting from. In other sports such as curling, where a partner’s goal impacts the behaviours of the other partner in meaningful ways (requiring less or more force), there may be both intentional and unintentional partner effects at work. Some researchers suggest that these contagion behaviours could be alleviated through self-focused psychological strategies, such as self-talk^50^ or through dyadic practice under conditions where partners have conflicting tasks, to help inhibit automatic imitation tendencies^51,52^). Further research is needed to confirm or refute such modifying effects.

Our study, while informative regarding the nature of motor contagion in novices, is not without limitations. For instance, we assessed only short-term practice and retention of the putting skill. Although our protocol conformed with previous investigations of dyad learning^19,21,22^, it could be that potential benefits from partner induced variability may become more evident with greater exposure (e.g., more trials) and after longer retention periods when compared to alone practice. Further, although we emphasize the important role of kinematics for inducing compensatory behaviours, in our putting task, differences in kinematics between putting to a near- and far-target may have been hard to distinguish. Ikegami et al.^17^ used a throwing task to investigate error-based contagions in experts, which as a result of the increased degrees of freedom involved in throwing, might better highlight differences in kinematics resulting in contagion type effects. Finally, we did not completely control for social facilitation, which may underpin some dyadic interaction effects, as individuals in the “alone” groups always had an experimenter present^33^. Although this procedure is congruent to methods used in previous dyad research^20,21,31^, in future research, other methods should be considered where the experimenter is removed or at least a less salient presence.

In conclusion, we have provided evidence of co-representation between novice learners, as evidenced by motor contagion behaviours in a golf putting task. Evidence of task-based contagion during practice was mostly independent of the practice conditions (simultaneous or alternating), suggesting that these behaviours were not dependent on observation of the partner’s kinematics. However, we did see some moderation of these effects coupled with predicted evidence of error-based compensation in the alternating dyads. We speculate that these effects are a result of prediction errors associated with seeing and evaluating a partner’s performance. There was little evidence that any contagion-type effects in practice impacted on measures of motor learning assessed in alone conditions the next day. Longer-term follow-up tests, as well as tests conducted both alone and in pairs, would allow stronger conclusions about potential benefits from practising with a partner. There is also a need for further research to separate the effects of different types of partner information (i.e., knowledge of goals, kinematics, and performance outcomes) and their contribution to motor skill learning, as well as additional testing of compensatory-type behaviours among novice performers, which are thought to be driven by prediction errors. Withholding feedback in later test phases may be one way to better determine longer-term partner-related effects, as well as to systematically vary partner-errors, through confederates that always overshoot or undershoot their assigned targets.

## Methods

### Study design

Participants were required to learn a golf putting skill to an assigned target, which was either near (150 cm) or far (300 cm), while practicing alone or with a novice partner. The study was conducted over two consecutive days; Day 1 comprised a familiarization phase, pre-test, practice phase and Day 2 comprised a retention test (same target distance as practice) and two transfer tests (opposite target distance as practice followed by a novel middle target distance) as illustrated in Fig. 1. Performance biases, characterised as task-based and error-based contagions, were assessed through constant error (CE), and overall performance was assessed through absolute error (AE) and variable error (VE).

### Participants

Ninety, right-handed participants between the ages of 18–37 years were tested (F = 59; M = 31). All participants had normal vision or wore corrective lenses, had no known neurological disorders, and provided informed consent before participating. Participants had little-to-no golf-related experience. Specifically, participants were asked to report the number of times they had played golf, gone to a driving range, putted mini golf, received golf lessons, or gained any other golfing experience within the previous 5 years and across their lifetime. The modal response was “0” times within the past 5-years (*M* = 0.53, *SD* = 1.49) and in their lifetime (*M* = 1.2, *SD* = 2.53). Participants were assigned to either an alone group (n = 30, F = 22), alternating dyad group (n = 30, F = 20: 15 dyads) or a simultaneous dyad group (n = 30, F = 18: 15 dyads). Assignment was quasi-random, with the constraint that another person of the same gender was available to be tested at the same time (for dyads). Within each of the three major groups, participants were randomly allocated to putt only to the near- or far-target during the practice phase (n = 15/target subgroup). Dyads always comprised same-gender partners, who had no previous relationship with their randomly assigned partner. The University of British Columbia’s Behavioural Research Ethics Board approved the study and guidelines of the university’s Behavioural Research Ethics Board were adhered to throughout the study.

### Task and apparatus

**Figure 7:**
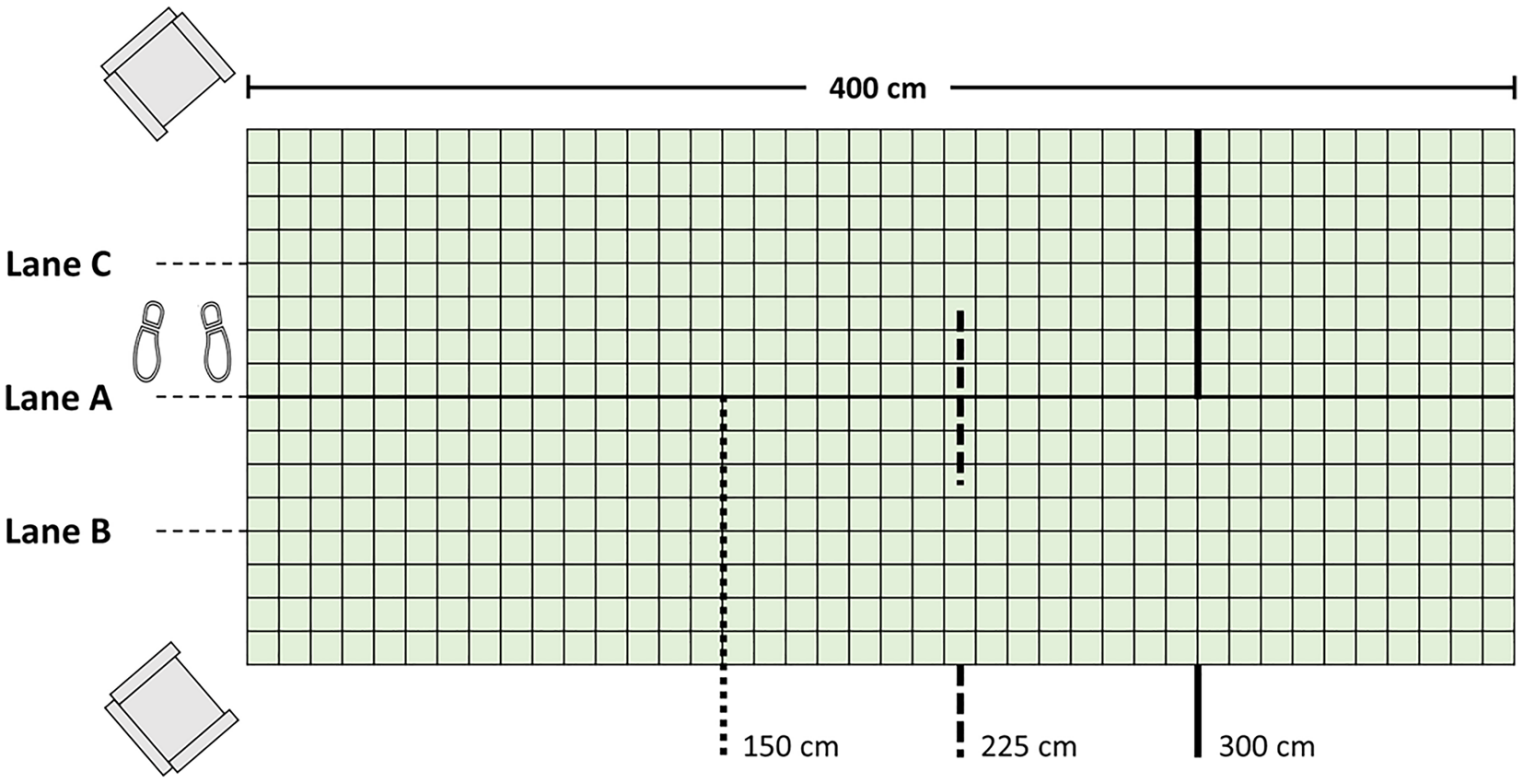
A schematic of the experimental set-up. Participants always putted from the same end of the grid (denoted by the feet icon). When putting alone participants putted in Lane A only. In contrast, partners in the two dyad groups putted in Lanes B and C, with alternating dyads taking turns and simultaneous dyads putting concurrently. The partner in Lane B always practiced to the near (150 cm) target, shown as the thick dotted line for illustration only, whereas the partner in Lane C always putted to the far (300 cm) target, shown as the thick black line for illustration only (targets were always denoted by a red diagonally positioned cross). Only for the transfer test did all participants putt to the middle (225 cm) target, shown as the thick dashed black line for illustration only. After each putt, participants sat on the chairs while the experimenter reported errors.

Using a “standard” (91 cm) right-handed golf club, participants were instructed to putt a golf ball (4.27 cm diameter) along a carpeted floor so that it stopped rolling as close to the centre of the target as possible. A chalked grid system was marked on the carpeted floor (280 cm wide × 450 cm long), in 10 cm^2^ grids, as illustrated in Fig. 7. Three red targets (2 cm in diameter) in the shape of crosses were conditionally placed either along the centre-line of the grid or on separate halves (lanes) of the grid (depending on group practice schedule; see Procedures). Targets were either 150 cm (near target), 225 cm (middle target), or 300 cm (far target) from the start line where the ball was placed. These targets were only visible to participants during the relevant experiment conditions.

### Materials

Participants first completed the Edinburgh Handedness Inventory to confirm individuals were right-hand dominant^53^. At the end of the first day after practice, participants completed golf putting appropriate modified versions of the Intrinsic Motivation Inventory (IMI^48^) and the User Engagement Scale (UES^49^). The IMI consisted of 22-items, comprising four subscales: interest/enjoyment (7-items), pressure/tension (5-items), perceived competence (5-items), and effort (5-items). We included an additional subscale, perceived partner’s competence (5-items), for the two dyad groups. Each item was answered on a 7-point Likert scale (1 = *not at all true–*7 = *very true*). The UES consisted of 25-items, comprising five subscales: perceived usability (7-items), novelty (3-items), felt involvement (4-items), focused attention (8-items), and endurability (3-items). Each item was answered on a 7-point, Likert scale (1 = *strongly disagree–*7 = *strongly agree*).

### Procedure

Upon entering the lab on Day 1, participants were given a consent form to read and complete as well as the handedness and golf experience questionnaires. Participants were informed about task goals, and standard instructions on how to hold and swing a golf club were provided alongside a demonstration (without hitting the ball).

All participants completed the familiarization and pre-test alone. Participants stood aside a chalked grid system as illustrated in Fig. 7 and performed three putts to familiarize themselves with the task (no targets present). Following familiarization, participants completed a 6-trial pre-test; 3 trials to the near-target (150 cm) and then 3 trials to the far-target (300 cm). Given that visual feedback was available during the pre-test and that this was a learning study, we restricted the number of these trials to ensure participants did not gain practice prior to our manipulations (for similar procedures see^20,22,54^). Participants’ errors (cm) in the x- and y-coordinates were recorded after each putt using the floor grid system and a tape measure. The distance was measured from the centre of the ball relative to the x- and y-coordinates of the centre of the target. Overshoots that exceeded the grid length were scored as + 50 cm in the y-direction beyond the grid length. For the pre-test, participants did not receive verbal feedback about their performance error. In the dyad groups, near-target partners performed the familiarization and pre-test first while their far-target partner completed the handedness and golf experience questionnaires in the hallway before switching.

During practice, participants performed 60 trials of putting to a single target (near or far). Before each trial, the experimenter would ask the participant if they were ready before slowly counting down “*three, two, one, go*”. Participants would hit the ball as the experimenter reached “*go*”. After each putt, participants would rest the golf club against the wall and have a seat in a chair facing the putting area. The experimenter then measured and recorded error from the centre of the target and verbally read-out the error scores back to the participant in order to provide specific feedback (overshoots and putts to the right of the target were positive values and undershoots and putts to the left of the target were negative values). The breaks in between trials were designed to keep overall practice duration relatively consistent across alone and dyad groups.

As illustrated in Fig. 7, partners in the dyad groups would putt on different halves of the grid (lanes B and C) with the alternating dyads taking turns and the simultaneous dyads putting concurrently. Both partners completed 60 physical practice trials each, consistently putting to their assigned target. During this practice phase, dyads were not permitted to communicate information about the task itself. Participants in the alternating dyad group would take turns observing their partner putt from the seat closest to their putting lane and then swap roles, sharing the same putter. The location of the chairs provided a side-on view of both their partner’s putting technique (kinematics) and the ball’s trajectory (outcome). After each attempt, participants always received the x-coordinate error before the y-coordinate error. The experimenter measured the error and the observing partner would record these measurements and repeat the errors back to the experimenter. This procedure was instigated to help keep partners attending to one another. For the simultaneous dyad group, after putting at the same time to their respective targets (in response to the experimenter’s count-down cue), participants took a seat and the experimenter then reported the error for each partner. Participants would again record their partner’s error and the order of delivery for these results would alternate by trial. Alone group participants also took a seat after each putt and recorded the feedback from the experimenter. Following the practice phase, all participants completed the motivation and engagement questionnaires.

On Day 2, approximately 24 h later, participants returned to the lab alone to complete a retention test followed by two transfer tests. During these tests, participants did not receive verbal feedback of their error but outcome feedback was not occluded. For the retention test, participants first performed ten trials to the same target that they had practiced. The first transfer test required participants to putt ten trials to the unassigned target during practice (i.e., the partner’s target). For the second 10-trial transfer test, participants putted to the “new” middle target (225 cm). Following the completion of the experiment, participants were fully debriefed and compensated for their time.

### Data analysis

Analyses were conducted in R^55^ using Linear Mixed Effects (LME) models. Two preplanned orthogonal Helmert contrasts were used to compare the alone group to the two dyad groups combined (contrast 1) and then to compare the two dyad groups separately (contrast 2), in lieu of an omnibus main effect test for partner-group. Target-group, whether individuals putted to the near- or far-target in practice, was another between-group fixed factor. For the pre-test and retention/transfer analyses, target (near vs. far, and also middle for transfer) was included as a repeated measures’ fixed factor. Time variables (i.e., practice trials) were aggregated into six blocks (10 trials/block). These blocks were treated as a continuous variable which was grand mean centered to reduce the likelihood of collinearity^56^. Fixed and random effect structures were determined through likelihood ratio tests using the Akaike information criterion^57^. Models were systematically built, first assessing the best fit for random effects (i.e., random intercepts and slopes across time variables for participants), before adding and testing fixed-effects (i.e., partner-group, target-group, and target). Fixed-effects were initially added independently and then with interactions. Where the interaction terms did not significantly improve model fit, models were simplified to only include main effects relevant to our primary hypotheses. When LME models did not account for random variance in the data, we ran fixed-effect linear regression analyses (i.e., methods adopted for traditional ANOVA), which provided the same output without random participant effects added. All tests were conducted with an a priori alpha of *p* < 0.05 denoting statistical significance. All model outputs are presented in supplementary materials.

### Outcome analyses

In view of hypotheses concerning partner errors based on target distance, analyses were limited to measurements in the y-direction. Measures were chosen to give indications of: 1) Constant (signed) Error (CE), relevant to bias in under or overshooting the target; 2) Absolute (unsigned) Error (AE), relevant to overall performance and learning; and 3) Variable Error (VE), relevant to between-trial variability within a block (i.e., SD of CE). Additional analyses of Radial Error (RE), which accounted for errors in both directions, are provided in supplementary materials. The pattern of data replicated those described for AE error in the y-direction.

Variable error:

$$\surd \left[ {\sum^{{\text{n}}}_{i} = {1 }\left( {{\text{CEy}}_{i} {-} \, \mu_{{{\text{CEy}}}} } \right)^{{2}} /n{-}{ 1}} \right]$$

For pre-test, retention, and transfer tests, we used omnibus LME models to test for statistical differences between subgroups. Therefore, this was a three-factor model including the fixed effects of Partner-group (Alone, Dyad-Alternating, and Dyad-Simultaneous), Target-group (near- and far-target), and Target (near and far for pre-test and retention; near, middle, and far for transfer), with repeated measures on this last factor. These analyses were conducted on AE and VE. We did not run these analyses on CE data, given our interest in learning-related effects rather than bias. For the practice data, a similar three factor LME analysis was conducted on AE and VE with practice block (B1-B6) as the repeated measures factor. For CE, because this measure is sensitive to the direction of error and we expected overshoot errors for the near-target groups, but undershoot errors for the far-target groups, we conducted separate LME analyses for near- and far-target subgroups.

To ascertain partner interdependence and to test for evidence of trial-to-trial compensation^58^ we ran between-trial correlations and analyses on the CE practice data. The specific model we used for analysis was the stability-influence model^59^. This model is essentially a 1-trial cross-lag correlation of each partner’s trial error on their partner’s next trial error (partner effect). The partner effect is considered an independent variable in the model allowing us to determine how a partner’s previous trial’s error impacts on their partner’s next trial. The first trial for all participants was removed, ensuring all trials were preceded by a partner observation. Partner independent variables were grand mean centered^59^.

We conducted a between-groups’ LME model to compare partner effects for the two dyad groups and for a pseudo “dyad-alone” group, where we yoked individuals from each alone subgroup based on participant number. The fixed-effects of partner-group and target-group were included as between-group factors. This model was compared to another including practice block (within-participant variable) to determine whether potential differences in partner effects were moderated by time. Comparison of models indicated that the addition of practice block did not improve model fit such that this factor was omitted.

### Psychosocial measures

Cronbach’s alpha values were calculated using the ltm package^60^ to assess internal consistencies for the subscales of each measure. For the Intrinsic Motivation Inventory (IMI), all Cronbach alpha values were good-to-excellent (all αs > 0.86). The internal consistency of the User Engagement Scale (UES) was acceptable-to-good for all subscales (all αs > 0.71). All psychosocial measures were analyzed using fixed-effect linear regression. Analyses were conducted on the independent subscales for each questionnaire.

### Supplementary Information

Supplementary Tables.

## Data Availability

The datasets generated during and/or analysed during the current study are available from the corresponding author on reasonable request.
