# Supplementary material for: Short-term evidence of partner-induced performance biases in simultaneous and alternating dyad practice in golf
Source: Sci Rep. 2023 Nov 30;13:21099. doi: 10.1038/s41598-023-48133-1 (PMC10689465; doi:10.1038/s41598-023-48133-1)
Supplement: Supplementary file 1 — Supplementary Tables. [file 41598_2023_48133_MOESM1_ESM.docx]

**Supplementary Materials**

Supplementary Table S1. Group means (M) in centimeters and standard deviations (SD) at pre-test for absolute error (AE), constant error (CE), and variable error (VE) in the y-direction.

|  |  | **Near** | | | | | |  | **Far** | | | | | |
| --- | --- | --- | --- | --- | --- | --- | --- | --- | --- | --- | --- | --- | --- | --- |
| **Target-group** | **Partner-Group*** | **AE** | | **CE** | | **VE** | |  | **AE** | | **CE** | | **VE** | |
|  |  | *M* | *SD* | *M* | *SD* | *M* | *SD* |  | *M* | *SD* | *M* | *SD* | *M* | *SD* |
| Near-target | Alone | 88.71 | 84.47 | 68.89 | 101.64 | 89.29 | 47.72 |  | 78.78 | 61.41 | 46.02 | 89.18 | 78.18 | 29.27 |
|  | Alternating | 117.32 | 111.22 | 97.21 | 129.55 | 105.89 | 62.97 |  | 90.14 | 69.12 | 61.01 | 96.34 | 80.16 | 49.99 |
|  | Simultaneous | 76.60 | 97.92 | 54.4 | 92.98 | 80.99 | 48.45 |  | 97.29 | 71.00 | 73.29 | 96.06 | 89.28 | 50.11 |
| Far-target | Alone | 105.07 | 107.43 | 93.33 | 117.99 | 58.81 | 36.44 |  | 106.81 | 76.77 | 88.43 | 97.79 | 89.46 | 46.84 |
|  | Alternating | 106.42 | 97.92 | 89.18 | 114.19 | 104.11 | 59.82 |  | 78.87 | 65.70 | 49.84 | 90.21 | 84.22 | 32.16 |
|  | Simultaneous | 75.91 | 76.86 | 53.02 | 94.48 | 66.09 | 35.59 |  | 98.60 | 68.84 | 61.36 | 104.08 | 82.80 | 39.93 |

____________________________________________________________________________________________________________

* No partner-group manipulation had taken place in the pre-test. We ran statistical comparisons here, in line with other data in the main body of the manuscript, to test for any pre-existing group differences that might obscure the practice data. For AE (as shown in Table 2, Supplementary analysis), there were no partner-group or target-group differences (*p*s > .37). There were also no differences in error when participants putted to the near- and far-targets (*p* = .59). There was, however, an interaction between the two dyad groups with target (*β* = -49.04 cm, *p* = .001). Alternating dyads had more error than the simultaneous dyads for the near-target, but they did not differ for the far-target. Dyads and alone groups did not differ. The constant error (CE) and variable error (VE) data mirrored these effects (see Supplementary Table 2).

Supplementary Table S2. Analysis of all pre-test performance data for absolute, constant, and variable error in the y-direction.

|  | **AE** | | | **CE** | | | **VE** | | |
| --- | --- | --- | --- | --- | --- | --- | --- | --- | --- |
| *Predictors* | *β* (cm) | *CI* | *p* | *β* (cm) | *CI* | *p* | *β* (cm) | *CI* | *p* |
| (Intercept) | 87.60 | 78.97 – 96.23 | **<.001** | 65.89 | 55.26 – 76.51 | **<.001** | 84.11 | 77.05 – 91.17 | **<.001** |
| Group (Dyads vs. Alone) | 1.73 | -16.58 – 20.03 | .85 | 7.61 | -14.92 – 30.14 | .51 | -7.76 | -22.73 – 7.21 | .31 |
| Group (Simultaneous vs Alternating) | 9.61 | -11.53 – 30.75 | .37 | 12.71 | -13.31 – 38.72 | .34 | 13.80 | -3.49 – 31.09 | .12 |
| Target-group (far-target vs. near-target) | 5.08 | -12.19 – 22.34 | .56 | 5.72 | -15.53 – 26.96 | .60 | -6.38 | -20.50 – 7.73 | .37 |
| Target (far vs. near) | -3.26 | -15.30 – 8.78 | .60 | -12.68 | -28.30 – 2.94 | .11 | -.18 | -12.49 – 12.13 | .98 |
| Group (Dyads vs. Alone)*Target (far vs. near) | -1.25 | -26.79 – 24.29 | .92 | -1.81 | -34.94 – 31.32 | .92 | 14.93 | -11.18 – 41.04 | .26 |
| Group (Simultaneous vs Alternating)*Target (far vs. near) | -49.04 | -78.53 – -19.56 | **.001** | -51.38 | -89.63 – -13.12 | **.009** | -35.31 | -65.46 – -5.16 | **.02** |
| **Random Effects** | | | | | | | | | |
| σ^2^ | 5068.98 | | | 8532.42 | | | 1749.40 | | |
| τ_00_ | 998.02 _Participant_ | | | 1246.28 _Participant_ | | | 275.83 _Participant_ | | |
| τ_11_ | 598.80 _Participant.Trial.c_ | | | 661.67 _Participant.Trial.c_ | | |  | | |
| ρ_01_ | -.51 _Participant_ | | | -.31 _Participant_ | | |  | | |
| ICC | .16 | | | .13 | | | .14 | | |
| N | 90 _Participant_ | | | 90 _Participant_ | | | 90 _Participant_ | | |
| Observations | 540 | | | 540 | | | 180 | | |
| Marginal R^2^ / Conditional R^2^ | .020 / .181 | | | .020 / .145 | | | .056 / .185 | | |

AE = Absolute error; VE = Variable error; CE = Constant error; *CI* = confidence intervals

Supplementary Table S3. Group means (M) in centimeters and standard deviations (SD) at acquisition for constant error in the y-direction.

| **Target-group** | **Partner-Group** | **Block 1** | | **Block 2** | | **Block 3** | | **Block 4** | | **Block 5** | | **Block 6** | | **Overall** | |
| --- | --- | --- | --- | --- | --- | --- | --- | --- | --- | --- | --- | --- | --- | --- | --- |
|  |  | *M* | *SD* | *M* | *SD* | *M* | *SD* | *M* | *SD* | *M* | *SD* | *M* | *SD* | *M* | *SD* |
| Near-target | Alone | 13.18 | 50.20 | 8.46 | 47.13 | 7.00 | 42.77 | 8.41 | 41.88 | .14 | 38.30 | 5.59 | 37.49 | 7.13 | 43.26 |
|  | Alternating | 22.08 | 60.25 | 22.29 | 60.26 | 7.91 | 46.76 | 11.02 | 46.26 | 20.18 | 50.93 | 11.69 | 48.50 | 15.86 | 52.67 |
|  | Simultaneous | 38.42 | 65.50 | 21.93 | 57.93 | 16.67 | 47.81 | 15.21 | 53.08 | 18.93 | 48.58 | 13.99 | 43.85 | 20.86 | 53.77 |
| Far-target | Alone | 22.32 | 75.19 | 9.53 | 69.71 | 9.53 | 64.02 | 2.45 | 58.55 | 11.25 | 66.32 | 7.66 | 62.35 | 10.49 | 66.32 |
|  | Alternating | 5.71 | 86.42 | 13.21 | 80.94 | 13.12 | 80.31 | 11.88 | 74.20 | 5.15 | 58.42 | 19.14 | 72.70 | 11.26 | 75.95 |
|  | Simultaneous | 20.31 | 79.32 | 9.32 | 67.60 | 18.07 | 63.62 | 12.78 | 68.50 | 5.95 | 65.29 | 7.63 | 63.44 | 12.34 | 68.19 |

Supplementary Table S4. Analysis outputs for all near-target subgroups’ constant error practice data in the y-direction.

|  | **CE** | | |
| --- | --- | --- | --- |
| *Predictors* | *β* (cm) | *CI* | *p* |
| (Intercept) | 14.62 | 10.58 – 18.66 | **<.001** |
| Practice Block | -4.05 | -6.03 – -2.07 | **<.001** |
| Group (Dyads vs. Alone) | -11.23 | -19.79 – -2.66 | **.010** |
| Group (Simultaneous vs. Alternating) | -4.98 | -14.88 – 4.91 | .32 |
| **Random Effects** | | | |
| σ^2^ | 2334.34 | | |
| τ_00_ | 152.00 _Participant_ | | |
| τ_11_ | 6.97 _Participant.Block.c_ | | |
| ρ_01_ | -.00 _Participant_ | | |
| ICC | .06 | | |
| N | 45 _Participant_ | | |
| Observations | 2700 | | |
| Marginal R^2^ / Conditional R^2^ | .019 / .082 | | |

CE = Constant error; *CI* = confidence intervals

Supplementary Table S5. Analysis outputs for all far-target subgroups’ constant error practice data in the y-direction.

|  | **CE** | | |
| --- | --- | --- | --- |
| *Predictors* | *β* (cm) | *CI* | *p* |
| (Intercept) | 11.36 | 7.20 – 15.52 | **<.001** |
| Practice block | -1.80 | -4.42 – .81 | .18 |
| Group (Dyads vs. Alone) | -1.31 | -10.14 – 7.52 | .77 |
| Group (Simultaneous vs. Alternating) | -1.09 | -11.28 – 9.10 | .83 |
| **Random Effects** | | | |
| σ^2^ | 4808.16 | | |
| τ_00_ | 122.51 _Participant_ | | |
| τ_11_ |  | | |
| ρ_01_ |  | | |
| ICC | .02 | | |
| N | 45 _Participant_ | | |
| Observations | 2700 | | |
| Marginal R^2^ / Conditional R^2^ | .001 / .026 | | |

CE = Constant error; *CI* = confidence intervals

Supplementary Table S6. Omnibus analysis of practice data (absolute and variable error) in the y-direction.

|  | **AE** | | | **VE** | | |
| --- | --- | --- | --- | --- | --- | --- |
| *Predictors* | *β* (cm) | *CI* | *p* | *β* (cm) | *CI* | *p* |
| (Intercept) | 39.34 | 35.90 – 42.77 | **<.001** | 45.44 | 41.91 – 48.98 | **<.001** |
| Practice Block | -4.08 | -5.41 – -2.75 | **<.001** | -4.89 | -6.47 – -3.30 | **<.001** |
| Group (Dyads vs. Alone) | -3.99 | -9.03 – 1.06 | .12 | -4.62 | -9.70 – .47 | .07 |
| Group (Simultaneous vs. Alternating) | .69 | -5.13 – 6.52 | .81 | 3.04 | -2.83 – 8.91 | .31 |
| Target-group (Far-target vs. Near-target) | 15.49 | 10.73 – 20.24 | **<.001** | 20.60 | 15.80 – 25.39 | **<.001** |
| **Random Effects** | | | | | | |
| σ^2^ | 1473.66 | | | 213.36 | | |
| τ_00_ | 119.49 _Participant_ | | | 122.07 _Participant_ | | |
| τ_11_ | 16.89 _Participant.Block.c_ | | | 23.07 _Participant.Block.c_ | | |
| ρ_01_ | -.49 _Participant_ | | | -.70 _Participant_ | | |
| ICC | .08 | | | .40 | | |
| N | 90 _Participant_ | | | 90 _Participant_ | | |
| Observations | 5400 | | | 540 | | |
| Marginal R^2^ / Conditional R^2^ | .047 / .13 | | | .28 / .57 | | |

AE = Absolute error; VE = Variable error; *CI* = confidence intervals

Supplementary Table S7. Partner-based trial-to-trial error analysis of practice data.

|  | **CE** | | |
| --- | --- | --- | --- |
| *Predictors* | *β* (cm) | *CI* | *p* |
| (Intercept) | 12.20 | 8.95 – 15.44 | **<.001** |
| Group (Dyads vs. Alone) | -5.86 | -12.94 – 1.22 | .102 |
| Group (Simultaneous vs. Alternating) | -2.78 | -10.95 – 5.40 | .50 |
| Target-group (far-target vs. near-target) | 2.02 | -.97 – 5.02 | .19 |
| Partner Effect | -.01 | -.04 – .02 | .57 |
| Group (Dyads vs. Alone)*Target-group (far-target vs. near-target) | -5.77 | -12.13 – .58 | .08 |
| Group (Simultaneous vs. Alternating)*Target-group (Far-target | -1.85 | -9.19 – 5.49 | .62 |
| Group (Dyads vs. Alone)*Partner Effect | .05 | -.02 – .12 | .17 |
| Group (Simultaneous vs. Alternating)*Partner Effect | -.12 | -.19 – -.04 | **.002** |
| Target-group*Partner Effect | .02 | -.01 – .06 | .19 |
| Group (Dyads vs. Alone)*Target-group*Partner Effect | -.09 | -.17 – -.01 | **.02** |
| Group (Simultaneous vs. Alternating)*Target-group*Partner Effect | .08 | -.01 – .16 | .07 |
| **Random Effects** | | | |
| σ^2^ | 2272.58 | | |
| τ_00_ _Dyad_ | 153.05 | | |
| τ_11_ _Dyad.P1_dum_ | 292.24 | | |
| τ_11_ _Dyad.P2_dum_ | .31 | | |
| τ_11_ _Dyad.P1_dum:Cent_Prt_ | . | | |
| τ_11_ _Dyad.P2_dum:Cent_Prt_ | .01 | | |
| ρ_01_ | -.64 | | |
|  | .63 | | |
|  | .45 | | |
|  | -.68 | | |
| ICC | .06 | | |
| N _Dyad_ | 45 | | |
| Observations | 5310 | | |
| Marginal R^2^ / Conditional R^2^ | .02 / .08 | | |

CE = Constant error; *CI* = confidence intervals

Supplementary Table S8. Omnibus analysis of target specific absolute and variable error (y-direction) for all dyad groups and subgroups.

|  | **AE** | | | **VE** | | |
| --- | --- | --- | --- | --- | --- | --- |
| *Predictors* | *β* (cm) | *CI* | *p* | *β* (cm) | *CI* | *p* |
| (Intercept) | 43.76 | 41.50 – 46.02 | **<.001** | 53.49 | 50.88 – 56.09 | **<.001** |
| Group (Dyads vs. Alone) | -.77 | -5.56 – 4.02 | .75 | -.09 | -5.61 – 5.43 | .97 |
| Group (Simultaneous vs. Alternating) | -1.76 | -7.29 – 3.77 | .53 | -1.91 | -8.28 – 4.47 | .56 |
| Target-group (far-target vs. near-target) | .73 | -3.79 – 5.24 | .75 | 1.66 | -3.55 – 6.86 | .53 |
| Target (middle vs. near) | 1.80 | -1.51 – 5.11 | .29 | .62 | -3.62 – 4.87 | .77 |
| Target (far vs. near) | 19.96 | 16.65 – 23.27 | **<.001** | 21.73 | 17.49 – 25.98 | **<.001** |
| Target-group (far-target vs. near-target)*Target (middle vs. near) | .64 | -5.98 – 7.26 | .85 | -1.41 | -9.90 – 7.08 | .74 |
| Target-group (far-target vs. near-target)*Target (far vs. near) | 13.13 | 6.51 – 19.76 | **<.001** | 14.98 | 6.49 – 23.47 | **.001** |
| Group (Dyads vs. Alone)*Target (middle vs. near) | 4.39 | -2.63 – 11.42 | .22 |  |  |  |
| Group (Simultaneous vs. Alternating)*Target (middle vs. near) | 2.71 | -5.40 – 10.82 | .51 |  |  |  |
| Group (Dyads vs. Alone)*Target (far vs. near) | 7.82 | .80 – 14.84 | **.029** |  |  |  |
| Group (Simultaneous vs. Alternating)*Target (far vs. near) | -.18 | -8.29 – 7.93 | .97 |  |  |  |
| **Random Effects** | | | | | | |
| σ^2^ | 1283.01 | | | 209.33 | | |
| τ_00_ | 79.04 _Participant_ | | | 87.48 _Participant_ | | |
| τ_11_ | 32.90 _Participant.Trial.c_ | | |  | | |
| ρ_01_ | -.27 _Participant_ | | |  | | |
| ICC | .06 | | | .29 | | |
| N | 90 _Participant_ | | | 90 _Participant_ | | |
| Observations | 2700 | | | 270 | | |
| Marginal R^2^ / Conditional R^2^ | .064 / .119 | | | .284 / .495 | | |

AE = Absolute error; VE = Variable error; *CI* = confidence intervals

Supplementary Table S9. Comparisons of Partner-group (ALT = Alternating and SIM = Simultaneous) and Target-group for the Intrinsic Motivation Inventory (IMI) subscales.

|  | **Interest/**  **enjoyment** | | **Perceived competence** | | **Partner competence** | | **Pressure** | | **Effort** | |
| --- | --- | --- | --- | --- | --- | --- | --- | --- | --- | --- |
| *Predictors* | *β* | *CI* | *β* | *CI* | *β* | *CI* | *β* | *CI* | *β* | *CI* |
| Intercept | 4.51*** | 4.13 – 4.89 | 3.50*** | 3.19 – 3.81 | 4.76*** | 4.19 – 5.33 | 3.05*** | 2.67 – 3.44 | 4.89*** | 4.52 – 5.26 |
| Group (Dyads vs. Alone) | .70 | -.11 – 1.51 | .59 | -.08 – 1.25 | - | | .72 | -.10 – 1.54 | .32 | -.46 – 1.10 |
| Group (SIM vs. ALT) | -.20 | -1.13 – .73 | -.13 | -.90 – .63 | .17 | -.63 – .97 | .21 | -.73 – 1.16 | .11 | -.80 – 1.01 |
| Target-group (far-target vs. near-target) | -.21 | -.75 – .33 | .39 | -.06 – .83 | -.53 | -1.33 – .27 | -.32 | -.86 – .23 | .26 | -.26 – .78 |
| Group (Dyads vs. Alone)* Target-group (far-target vs. near-target) | -1.41* | -2.56 – -.27 | -1.30** | -2.24 – -.36 | - | | -.91 | -2.07 – .25 | -.17 | -1.28 – .94 |
| Group (SIM vs. ALT)* Target-group (far-target vs. near-target) | -.85 | -2.16 – .47 | -.81 | -1.90 – .27 | -.23 | -1.36 – .91 | -.32 | -1.66 – 1.02 | -.65 | -1.93 – .63 |
| Observations | 90 | | 90 | | 60 | | 90 | | 90 | |
| R^2^ / R^2^ adjusted | .12 / .07 | | .17 / .12 | | .09 / .04 | | .054 / -.002 | | .04 / -.02 | |

*CI* = confidence intervals; * = *p* < 0.05; ** = *p* < 0.01; *** = *p* < 0.001

Supplementary Table S10. Comparison of Partner-group (ALT = Alternating and SIM = Simultaneous) and Target-group for the User Engagement Scale (UES) subscales.

|  | **Usability** | | **Novelty** | | **Involvement** | | **Attention** | | **Endurability** | |
| --- | --- | --- | --- | --- | --- | --- | --- | --- | --- | --- |
| *Predictors* | *β* | *CI* | *β* | *CI* | *β* | *CI* | *β* | *CI* | *β* | *CI* |
| (Intercept) | 5.30******* | 5.01 – 5.59 | 4.65******* | 4.23 – 5.07 | 4.84******* | 4.48 – 5.20 | 3.86******* | 3.49 – 4.22 | 4.31******* | 3.95 – 4.67 |
| Group (Dyads vs. Alone) | -.29 | -.90 – .32 | .42 | -.46 – 1.31 | -.08 | -.85 – .69 | -.03 | -.81 – .75 | .40 | -.37 – 1.17 |
| Group (SIM vs. ALT) | -.30 | -1.00 – .41 | -.09 | -1.11 – .93 | -.10 | -.99 – .79 | -.35 | -1.25 – .55 | .00 | -.89 – .89 |
| Target-group (far-target vs. near-target) | .09 | -.32 – .49 | -.31 | -.90 – .28 | -.11 | -.62 – .41 | -.23 | -.75 – .29 | .06 | -.45 – .57 |
| Group (Dyads vs. Alone)* Target-group (far-target vs. near-target) | .04 | -.82 – .91 | -1.03 | -2.28 – .22 | -.44 | -1.53 – .65 | -.72 | -1.83 – .38 | -1.09* | -2.17 – -.00 |
| Group (SIM vs. ALT)* Target-group (far-target vs. near-target) | .01 | -.99 – 1.01 | -1.04 | -2.49 – .40 | -1.02 | -2.27 – .24 | .04 | -1.23 – 1.32 | -.84 | -2.10 – .41 |
| Observations | 90 | | 90 | | 90 | | 90 | | 90 | |
| R^2^ / R^2^ adjusted | .04 / -.02 | | .10 / .04 | | .09 / .04 | | .06 / .006 | | .09 / .03 | |

*CI* = confidence intervals; * = *p* < 0.05; ** = *p* < 0.01; *** = *p* < 0.001

Supplementary Table S11. Group means (M) and standard deviations (SD) at pre-test for radial error in centimeters.

| **Target-group** | **Partner-group** | **Near** | |  | **Far** | |
| --- | --- | --- | --- | --- | --- | --- |
|  |  | *M* | *SD* |  | *M* | *SD* |
| Near-target | Alone | 90.29 | 83.72 |  | 81.02 | 59.94 |
|  | Alternating | 118.19 | 110.99 |  | 91.84 | 67.65 |
|  | Simultaneous | 78.08 | 74.74 |  | 101.15 | 69.51 |
| Far-target | Alone | 106.27 | 106.81 |  | 110.57 | 73.21 |
|  | Alternating | 107.28 | 97.59 |  | 81.42 | 63.84 |
|  | Simultaneous | 78.34 | 75.53 |  | 103.25 | 65.55 |

Supplementary Table S12. Group means (M) and standard deviations (SD) at each practice block for radial error in centimeters.

| **Target-group** | **Partner-group** | **Block 1** | |  | **Block 2** | |  | **Block 3** | |  | **Block 4** | |  | **Block 5** | |  | **Block 6** | |
| --- | --- | --- | --- | --- | --- | --- | --- | --- | --- | --- | --- | --- | --- | --- | --- | --- | --- | --- |
|  |  | *M* | *SD* |  | *M* | *SD* |  | *M* | *SD* |  | *M* | *SD* |  | *M* | *SD* |  | *M* | *SD* |
| Near-target | Alone | 40.40 | 33.50 |  | 37.99 | 30.05 |  | 36.60 | 24.24 |  | 36.03 | 24.22 |  | 33.44 | 19.99 |  | 31.18 | 22.56 |
|  | Alternating | 47.15 | 43.99 |  | 48.36 | 43.09 |  | 38.50 | 28.59 |  | 37.99 | 29.48 |  | 39.15 | 38.88 |  | 37.12 | 33.94 |
|  | Simultaneous | 57.27 | 51.38 |  | 46.77 | 42.23 |  | 40.79 | 31.25 |  | 43.82 | 34.68 |  | 43.68 | 30.16 |  | 36.95 | 28.83 |
| Far-target | Alone | 66.74 | 43.75 |  | 57.27 | 43.54 |  | 55.73 | 36.11 |  | 50.79 | 32.22 |  | 57.96 | 37.87 |  | 53.96 | 35.93 |
|  | Alternating | 67.17 | 55.95 |  | 63.51 | 54.03 |  | 65.49 | 49.93 |  | 61.31 | 45.38 |  | 47.96 | 36.75 |  | 56.51 | 50.87 |
|  | Simultaneous | 71.57 | 46.96 |  | 60.25 | 39.35 |  | 59.84 | 37.04 |  | 59.60 | 41.98 |  | 57.08 | 38.25 |  | 55.45 | 38.83 |

Supplementary Table S13. Group means (M) and standard deviations (SD) at retention and transfer testing for radial error in centimeters.

| **Target-group** | **Partner-group** | **Near** | |  | **Middle** | |  | **Far** | |
| --- | --- | --- | --- | --- | --- | --- | --- | --- | --- |
|  |  | *M* | *SD* |  | *M* | *SD* |  | *M* | *SD* |
| Near-target | Alone | 38.44 | 28.19 |  | 37.47 | 29.68 |  | 54.26 | 39.10 |
|  | Alternating | 44.46 | 30.09 |  | 40.32 | 35.63 |  | 55.81 | 46.62 |
|  | Simultaneous | 44.42 | 32.06 |  | 47.24 | 40.34 |  | 56.60 | 43.14 |
| Far-target | Alone | 41.66 | 27.22 |  | 34.74 | 30.92 |  | 69.99 | 47.29 |
|  | Alternating | 37.48 | 23.63 |  | 39.07 | 31.19 |  | 59.67 | 48.81 |
|  | Simultaneous | 37.80 | 23.99 |  | 39.91 | 32.78 |  | 67.07 | 66.17 |

Supplementary Table S14. Comparison of partner and target groups’ radial error (RE) at pre-test.

|  | **RE** | | |
| --- | --- | --- | --- |
| *Predictors* | *β* (cm) | *CI* | *p* |
| (Intercept) | 87.88 | 74.49 – 101.27 | **<.001** |
| Group (Dyads vs. Alone) | 2.26 | -19.70 – 24.21 | .84 |
| Group (Simultaneous vs. Alternating) | 33.09 | 7.74 – 58.44 | **.01** |
| Target-group (far-target vs. near-target) | 5.64 | -11.34 – 22.63 | .51 |
| Target (far vs. near) | -1.54 | -13.36 – 10.29 | .79 |
| Group (Dyads vs. Alone)* Target (far vs. near) | -1.43 | -26.52 – 23.65 | .91 |
| Group (Simultaneous vs. Alternating)* Target (far vs. near) | -50.10 | -79.06 – -21.13 | **.001** |
| **Random Effects** | | | |
| σ^2^ | 4891.58 | | |
| τ_00_ _Participant_ | 968.87 | | |
| τ_11_ _Participant.Trial.c_ | 604.28 | | |
| ρ_01_ _Participant_ | -.50 | | |
| ICC | .17 | | |
| N _Participant_ | 90 | | |
| Observations | 540 | | |
| Marginal R^2^ / Conditional R^2^ | .02 / .18 | | |

Supplementary Table S15. Omnibus analysis of radial error (RE) during practice for partner and target-groups.

|  | **RE** | | |
| --- | --- | --- | --- |
| *Predictors* | *β* (cm) | *CI* | *p* |
| (Intercept) | 87.11 | 75.10 – 99.12 | **<.001** |
| Practice block | -4.13 | -5.42 – -2.84 | **<.001** |
| Group (Dyads vs. Alone) | -4.50 | -9.49 – .48 | .08 |
| Group (Simultaneous vs. Alternating) | -1.71 | -7.46 – 4.04 | .56 |
| Target-group (far-target vs. near-target) | 18.49 | 13.79 – 23.18 | **<.001** |
| **Random Effects** | | | |
| σ^2^ | 1363.84 | | |
| τ_00_ _Participant_ | 116.88 | | |
| τ_11_ _Participant.Block_ | 16.48 | | |
| ρ_01_ _Participant_ | -.46 | | |
| ICC | .09 | | |
| N _Participant_ | 90 | | |
| Observations | 5400 | | |
| Marginal R^2^ / Conditional R^2^ | .07 / .15 | | |

Supplementary Table S16. Radial error (RE) in retention and transfer testing on day 2

|  | **RE** | | |
| --- | --- | --- | --- |
| *Predictors* | *β* (cm) | *CI* | *p* |
| (Intercept) | 40.59 | 36.48 – 44.69 | **<.001** |
| Target-group (far-target vs. near-target) | -3.44 | -9.24 – 2.36 | .25 |
| Group (Dyads vs. Alone) | -5.03 | -11.18 – 1.13 | .11 |
| Group (Simultaneous vs. Alternating) | -4.51 | -11.61 – 2.60 | .21 |
| Target (middle vs. near) | .76 | -3.77 – 5.30 | .74 |
| Target (far vs. near) | 13.88 | 9.35 – 18.41 | **<.001** |
| Target-group (far-target vs. near-target)*Target (middle vs. near) | .31 | -6.10 – 6.72 | .92 |
| Target-group (far-target vs. near-target)*Target (far vs. near) | 13.45 | 7.05 – 19.86 | **<.001** |
| Group (Dyads vs. Alone)*Target (middle vs. near) | 4.53 | -2.27 – 11.33 | .19 |
| Group (Simultaneous vs. Alternating)*Target (middle vs. near) | 3.74 | -4.11 – 11.59 | .35 |
| Group (Dyads vs. Alone)*Target (far vs. near) | 7.36 | .57 – 14.16 | **.034** |
| Group (Alternating vs. Simultaneous)*Target (far vs. near) | -.22 | -8.07 – 7.63 | .96 |
| **Random Effects** | | | |
| σ^2^ | 1201.86 | | |
| τ_00_ _Participant_ | 79.59 | | |
| τ_11_ _Participant.Trial.c_ | 31.44 | | |
| ρ_01_ _Participant_ | -.28 | | |
| ICC | .06 | | |
| N _Participant_ | 90 | | |
| Observations | 2700 | | |
| Marginal R^2^ / Conditional R^2^ | .08 / .13 | | |
